# Periplasmic synthesis and purification of the human prolactin antagonist Δ_1-11_-G129R-hPRL

**DOI:** 10.1186/s13568-021-01209-5

**Published:** 2021-04-27

**Authors:** Miriam F. Suzuki, Larissa A. Almeida, Stephanie A. Pomin, Felipe D. Silva, Renan P. Freire, João E. Oliveira, Regina Affonso, Carlos R. J. Soares, Paolo Bartolini

**Affiliations:** Biotechnology Center, Instituto de Pesquisas Energéticas e Nucleares, IPEN-CNEN/SP, Avenida Prof. Lineu Prestes 2242, São Paulo, SP 05508-000 Brazil

**Keywords:** Prolactin antagonist, Periplasmic expression, DsbA signal peptide, Antagonistic properties

## Abstract

The human prolactin antagonist Δ_1-11_-G129R-hPRL is a 21.9 kDa recombinant protein with 188 amino acids that downregulates the proliferation of a variety of cells expressing prolactin receptors. Periplasmic expression of recombinant proteins in *E. coli* has been considered an option for obtaining a soluble and correctly folded protein, as an alternative to cytoplasmic production. The aim of this work was, therefore, to synthesize for the first time, the Δ_1-11_-G129R-hPRL antagonist, testing different activation temperatures and purifying it by classical chromatographic techniques. *E. coli* BL21(DE3) strain was transformed with a plasmid based on the pET25b( +) vector, DsbA signal sequence and the antagonist cDNA sequence. Different doses of IPTG were added, activating under different temperatures, and extracting the periplasmic fluid via osmotic shock. The best conditions were achieved by activating at 35 °C for 5 h using 0.4 mM IPTG, which gave a specific expression of 0.157 ± 0.015 μg/mL/A_600_ at a final optical density of 3.43 ± 0.13 A_600_. Purification was carried out by nickel-affinity chromatography followed by size-exclusion chromatography, quantification being performed via high-performance size-exclusion chromatography (HPSEC). The prolactin antagonist was characterized by SDS-PAGE, Western blotting, reversed-phase high-performance liquid chromatography (RP-HPLC) and MALDI-TOF–MS. The final product presented > 95% purity and its antagonistic effects were evaluated in vitro in view of potential clinical applications, including inhibition of the proliferation of cancer cells overexpressing the prolactin receptor and specific antidiabetic properties, taking also advantage of the fact that this antagonist was obtained in a soluble and correctly folded form and without an initial methionine.

## Introduction

Recombinant human prolactin antagonists are potential drugs that inhibit target prolactin receptors in dopamine-resistant prolactinomas, breast cancer, prostate cancer, and ovary cancer, in all cases where autocrine PRL acts as growth promoting agent, or even for pain release and to avoid hair loss (O'Sullivan and Bates 2016). There are several PRLR antagonists reported in the literature (Goffin 2017; Tallet et al. 2008) that downregulate the proliferation of a variety of cells expressing prolactin receptors: G129R-hPRL (Chen et al. 1999), S179D-hPRL (Chen et al. 1998), Δ_1-9-_G129R-hPRL and Δ_1-14_-G129R-hPRL (Bernichtein et al 2003a, b, c), Δ_1-9_-C11S-S33A-Q73L-G129R-K190R-hPRL (Yu et al. 2019) and also G120R-hGH (Menezes et al. 2017). All prolactin antagonists have been up to now synthesized as inclusion bodies in the cytoplasm of *E. coli* (Chen et al. 1998; Oclon et al. 2018; Yu et al. 2019), or secreted into the medium by transfected CHO cells (Soares et al. 2006; Swiech et al. 2012) or mouse L-cells (Chen et al. 1998). Periplasmic expression of recombinant proteins in *E. coli* has been considered to be a valid option for obtaining a soluble and correctly folded protein, as an alternative to the cytoplasmic production, in inclusion bodies, of an unfolded, insoluble protein carrying an extra initial methionine (Dalmora et al. 1997; Dias et al. 2018; Menezes et al. 2017; Morganti et al. 1998; Sockolosky and Szoka 2013; Suzuki et al. 2012; Taherian et al. 2019).

The importance of prolactin receptor antagonists is especially related to their potential to antagonize the tumor growth-promoting effects of hPRL in vivo, in animal models of breast and prostate cancer (Chen et al. 2002; Xu et al. 2001), and to their antiangiogenic properties (Ueda et al. 2006; 2009). More recently, they have been described as promising therapeutic agents to control blood glucose levels, showing antidiabetic properties (Capone et al. 2015; Furigo et al. 2019; Menezes et al. 2017).

Δ_1-11_-G129R-hPRL is a novel hPRL antagonist obtained by deleting eleven N-terminal residues of the well-known prolactin receptor antagonist G129R-hPRL. It is therefore a 21.9 kDa protein with 188 amino acids and pI 6.15. It has been reported that, by eliminating the first disulfide bond (C4-C11) forming the ring structure of the cystine knot, the protein loses any agonistic properties (Bernichtein et al. 2003a, b, c). The aim of this work was the expression of the Δ_1-11_-G129R-hPRL antagonist in the periplasm of *E. coli*, its purification and physical–chemical characterization and evaluation of its antagonistic effects using mouse lymphoblastic cells transfected with the human prolactin receptor (Bernichtein et al. 2003a, b, c). This homologous lactogenic assay (Ba/F3-LLP) is based on the Low Low cell Population (LLP) bioassay (Glezer et al. 2006), which has a sensitivity of the same order as the widely applied Nb2 bioassay and is about tenfold more sensitive than the original Ba/F3-LP assay (Bernichtein et al. 2003a, b, c; Paraiba et al. 2010).

## Materials and methods

### Strain and expression conditions

The *E. coli* BL21(DE3) strain was transformed with the pET25b( +) vector containing the DsbA (a bacterial disulfide oxireductase) signal sequence followed by the Δ_1-9_-G129R-hPRL antagonist cDNA and ampicillin resistance-sequences (database for human prolactin from NCBI reference sequence NP_000939.1 and consensus CDS 4548.1). Besides the substitution of the natural signal peptide by the DsbA signal peptide, the deletion of the initial 27 nucleotides and a substitution of glycine 129, codon GGC, by arginine, codon CGC, were also introduced. The plasmid was constructed using *NdeI* and *BamHI* restriction sites (Biomatik Custom Gene Synthesis Service, Cambridge, Ontario, Canada) without any His-tag sequence. After overnight culture at 37 °C, plasmids were extracted, analyzed by digestion to confirm the presence of the inserts, and sequenced by the Sanger method to confirm the correct DNA sequence. The BigDye Terminator v 3.1 Cycle Sequencing Kit was used, and amplicons were sequenced in an ABI 3730 DNA Analyzer (Life Technologies—Applied Biosystems/Hitachi, Foster City, CA, USA). All data were obtained via Sequencing Analysis 5.3.1.

The transformed bacteria were cultured in 100 mL Luria–Bertani broth (LB broth) with 0.1 mg/mL ampicillin, under rotational shaking (150 rpm) in 250 mL Erlenmeyer flasks. After overnight culture at 30 °C, recombinant protein production was evaluated at different temperatures (25, 30, 32, 35 and 37 °C) for 5 h, with different doses of IPTG (0.2; 0.4; 0.6; 0.8 and 1.0 mM). The periplasmic fluid was then obtained by osmotic shock.

### Osmotic shock

Periplasmic fluid was extracted after harvesting the bacteria by centrifugation at 3000 × g for 10 min as described (Sockolosky and Szoka 2013). Briefly, pellets were resuspended in hypertonic solution consisting of 10 mM Tris–HCl pH 7.5, adding 1 mL of 20% sucrose (w/v) and 33 µL 0.5 M EDTA pH 8.0 for each 100 A_600_ units. After 10 min in an ice bath, the bacteria were centrifuged at 3000 x *g* for 10 min. The pellet was resuspended with 1 mL of a hypotonic solution of 1 mM Tris–HCl pH 7.5 per each 100 A_600_ units, incubating then in an ice bath for 10 min. After centrifuging at 3000 x *g* for 10 min, the collected supernatant represented the periplasmic fluid, which was stored at − 80 °C, analyzed by SDS-PAGE, Western blotting and RP-HPLC.

### SDS-PAGE, Western blotting and RP-HPLC qualitative and quantitative analyses

15% Polyacrylamide gel electrophoresis (SDS-PAGE) was carried out under non-reducing conditions, staining with Coomassie brilliant blue G-250 (USB, Cleveland, OH, USA) (Laemmli 1970; Soares et al. 2003). Western blotting analysis was performed using semi-dry transfer on a nitrocellulose membrane. A rabbit anti-hPRL primary antibody (1:1,000) and a secondary HRP conjugated goat anti-rabbit Ig-G (1:10,000) were used (R&D Systems, Minneapolis, MN, USA) (Soares et al. 2003). The images were obtained by chemiluminescence (Immobilon Western Chemiluminescent HRP Substrate, Millipore Corporation, Billerica, MA, USA) using an UVITEC photo documenter System (Cambridge, United Kingdom) (Capone et al. 2015). RP-HPLC was used for the qualitative and quantitative analysis of this protein in all steps of purification, with a Shimadzu model SCL-10A HPLC apparatus coupled to a SPD-10AV UV detector with a Class VP software (Shimadzu, MD, USA) connected to a C4 Vydac 214TP54 column (25 cm × 4.6 mm ID, pore diameter of 300 Å and particle diameter of 5 µm, Hesperia, CA, USA), and a silica precolumn packed with LiChrosorb Si-60, 7.9–12.4 µm (Merck, Darmstadt, Germany), as described (Soares et al. 2002). The mobile phase consisted of 71% 50 mM Tris–HCl buffer, pH 7.5, and 29% n-propanol, with a flow rate of 0.5 mL/min, column temperature maintained at 45 °C, monitoring at 220 nm and applying a sample volume of 10–500 µL (Dalmora et al. 1997). The antagonist was quantified by determining the area under the curve against the Internal Standard of rec-hPRL and the International Standard of rec-hGH, coded WHO 98/574 (Soares et al. 2008).

### Purification process

Purification was carried out by automated nickel affinity chromatography followed by size exclusion chromatography (SEC) (Ueda et al. 2001). Before purification, a dialysis against 0.05 M sodium phosphate buffer, pH 7.2, was conducted to eliminate traces of EDTA, used for osmotic shock. The first purification step of Δ_1-11_-G129R-hPRL from the periplasmic fluid was conducted by nickel affinity chromatography (IMAC-HisPrep™ FF16/10, GE Healthcare Bio-Sciences AB, Uppsala, Sweden) with 0.02 M sodium phosphate, pH 7.2, 0.8 M NaCl as buffer A (flow rate 5 mL/min, particle size 90 µm, bed volume 20 mL, pressure limit of 0.5 MPa) and 100 mM imidazole as buffer B. After equilibrating the column with 0.3 M NiSO_4_, the dialyzed periplasmic fluid was injected onto the column and two steps of 10 mM and 20 mM imidazole (5 column volumes each) and one linear gradient of 20 mM to 100 mM imidazole (5 column volumes) were carried out. During this process, 5 mL fractions were collected. Considering the natural affinity of hPRL for nickel, no his-tag was introduced.

A pool of fractions containing the antagonist was then applied to a SEC column after concentration by centrifugation via an Amicon^®^ Ultra 15 (centrifugal filter devices, Merck Millipore Ltd., Tullagreen, Carrigtwohill, Co. Cork, Ireland) at 5000*g*, with fixed-angle rotor up to 40 mL. The resin was Sephacryl S-100, packed in a 26 × 100 mm column and running under isocratic conditions, with 0.02 M sodium phosphate buffer, pH 7.2, at a flow rate of 1 mL/min and pressure limit of 0.4 MPa. Fractions of 2 mL were collected and stored at − 80 °C.

In both cases, the purification was carried out using an ÄKTA purification system (GE Health Sciences, Buckinghamshire, UK) (Menezes et al. 2017; Silva et al. 2019).

### HPSEC and mass spectral characterization

Final quantification was carried out via HPSEC with the same Shimadzu apparatus, connected to a TosoHaas G2000 SW column (60 cm × 7.5 mm ID, particle size of 10 µm and pore size of 125 Å) coupled to a 7.5 × 7.5 mm ID SW guard column (Montgomeryville, PA, USA). The mobile phase was 0.025 M ammonium bicarbonate, pH 7.0, with a flow rate of 1.0 mL/min (Soares et al. 2002). The final product was also analyzed by MALDI-TOF MS. The exact molecular mass was determined by MALDI-TOF Autoflex Speed (Bruker Daltonics Inc., Billerica, MA, USA) using a sinapinic acid mixture (10 mg/mL TA30: 30% acetonitrile, 0.1% TFA): sample dilution 1:1 (v/v). The mixture was applied on a GroundSteel plate and analyzed between 10 and 30 kDa. The acquisition mode was linear with positive polarity (Protparam program) (Silva et al. 2019).

### N-terminal amino acid determination

The N-terminal amino acid sequence of the Δ_1-11_-G129R-hPRL was determined with the protein and peptide sequencer system (Model PPSQ-21, Shimadzu, Kyoto, Japan) through automated Edman Degradation, directly from a pure liquid sample at 100 µg/mL (Edman and Begg 1967).

### In vitro bioassay

The Ba/F3-LLP proliferation assay based on mouse lymphoblastic cells was applied to confirm the agonistic and antagonistic effects of this molecule (Menezes et al. 2017; Soares et al. 2006). These cells express human PRL receptor long isoform and have geneticin resistance. The maximum proliferation is obtained with 1 ng/mL of PRL in RPMI-1640 culture medium, supplemented with 10% heat inactivated FSB and 50 U/mL of penicillin, 50 µg/mL of streptomycin, 700 µg/mL of geneticin (G418, Sigma). Before the assay, the cells were starved for 12 h in RPMI-1640 medium containing 1% FBS. Cells were then distributed in a flat bottom 96 well-plate at a density of 2 × 10^4^ cells/well in a final volume of 100 µL of medium with 1% FBS and antibiotics. hPRL at concentration of 0.025 ng/mL to 1 ng/mL in 100 µL medium was added to each well for the standard curve. The antagonist alone, or with hPRL, was added in serial dilutions for proliferation evaluation. After 72 h at 37 °C and 5% CO_2_, the MTS assay was carried out as reported in the protocol (Promega Corp., Madison, WI, USA). After 2 h of incubation with 20 µL of the MTS/PMS mixture (v/v; 20:1), the absorbance at 490 nm was read in a microplate reader (Dynatech, model MR4000, Chantilly, VA, USA) (Soares et al. 2006). The recombinant hPRL International Standard (WHO-97/714), with a declared biological activity of 57.2 ± 11.4 IU/mg, was used for the standard curve (Paraiba et al. 2010).

### Statistical analyses

The results are expressed as mean ± standard deviation. Data were analyzed using one-way ANOVA and Tukey multi-comparisons. PRISM 6 (GraphPad, La Jolla, CA, USA.) software was used for the statistical analyses and only P values < 0.05 were considered to be statistically significant.

## Results

### Effects of temperature and IPTG concentration on the expression level

**Fig. 1:**
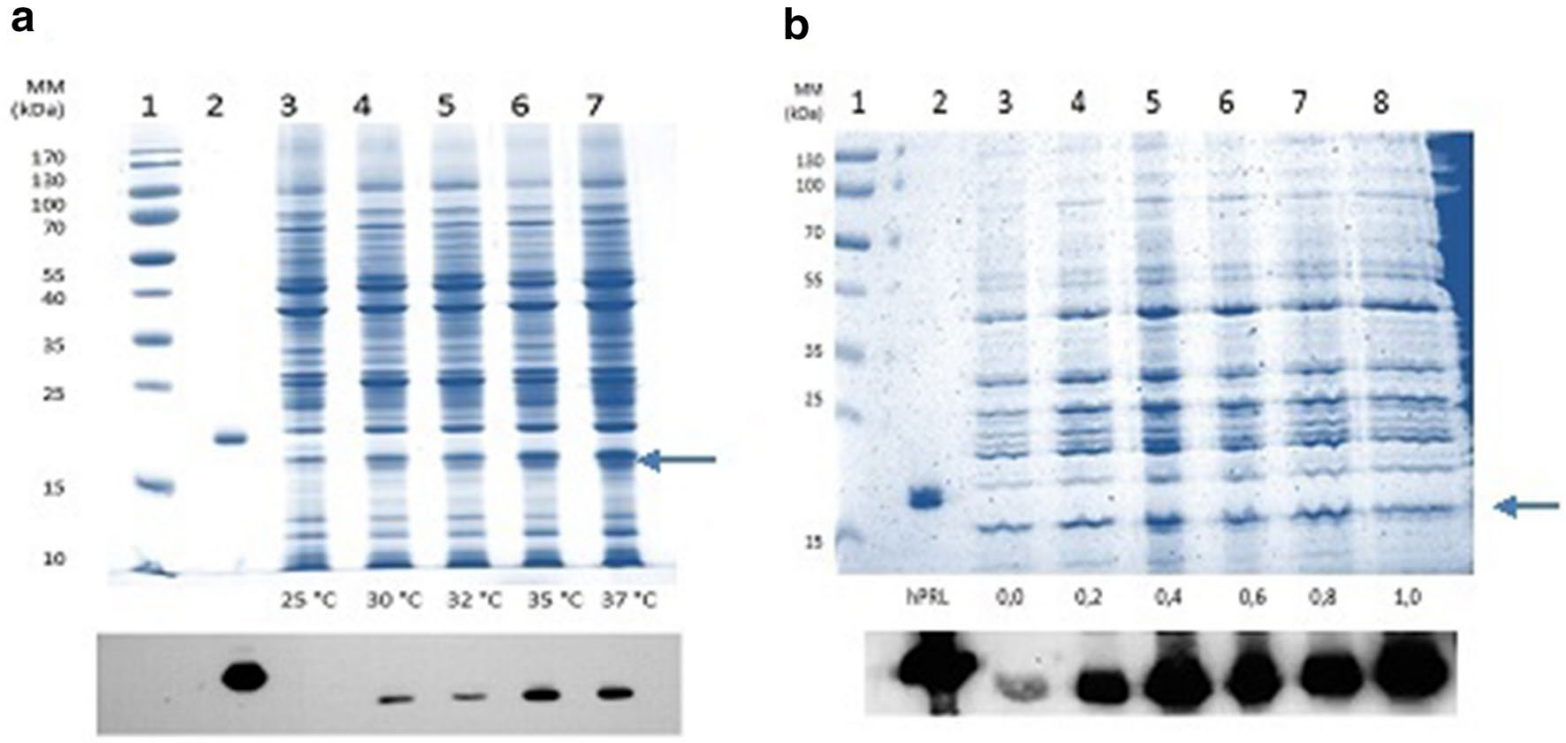
SDS-PAGE under non-reducing conditions and Western Blotting with samples of periplasmic fluid obtained from *E. coli* BL21(DE3) by osmotic shock, after 5 h of cultivation with 0.4 mM IPTG at different temperatures: 25 °C, 30 °C, 32 °C, 35 °C and 37 °C (**a**) and at 25 °C with different IPTG concentrations: 0.2 mM, 0.4 mM, 0.6 mM, 0.8 mM and 1.0 mM. **b** Lane 1: molecular mass marker, lane 2: recombinant human prolactin internal standard (23 kDa). The arrows indicate the position of Δ_1-11_-G129R-hPRL (22 kDa)

The best conditions for producing the Δ_1-11_-G129R-hPRL antagonist were confirmed at a temperature of 35 °C (Fig. 1a) for 5 h with 0.4 mM IPTG (Fig. 1b).

**Fig. 2:**
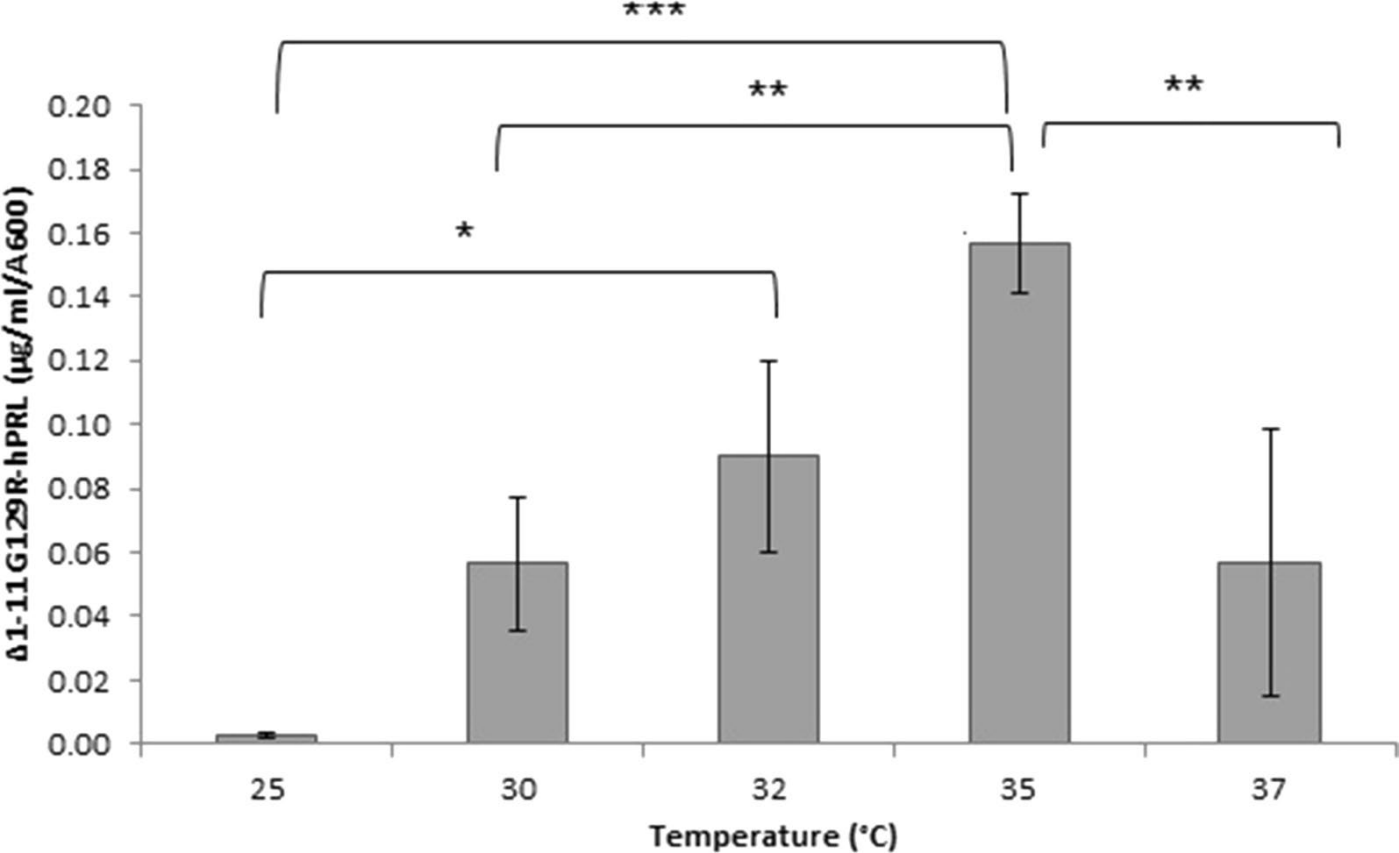
Influence of the temperature on Δ_1-11_-G129R-hPRL synthesis (n = 3) 5 h after addition of 0.4 mM IPTG to the *E. coli* BL21 (DE3) culture. *P < 0.05, **P < 0.01, ***P < 0.001

The periplasmic fluid was analyzed by RP-HPLC and a specific expression of 0.157 ± 0.015 μg/mL/A_600_, n = 3 (Fig. 2) with a final optical density of 3.43 ± 0.13 A_600_ (n = 3) was obtained in the Erlenmeyer incubation flasks.

**Fig. 3:**
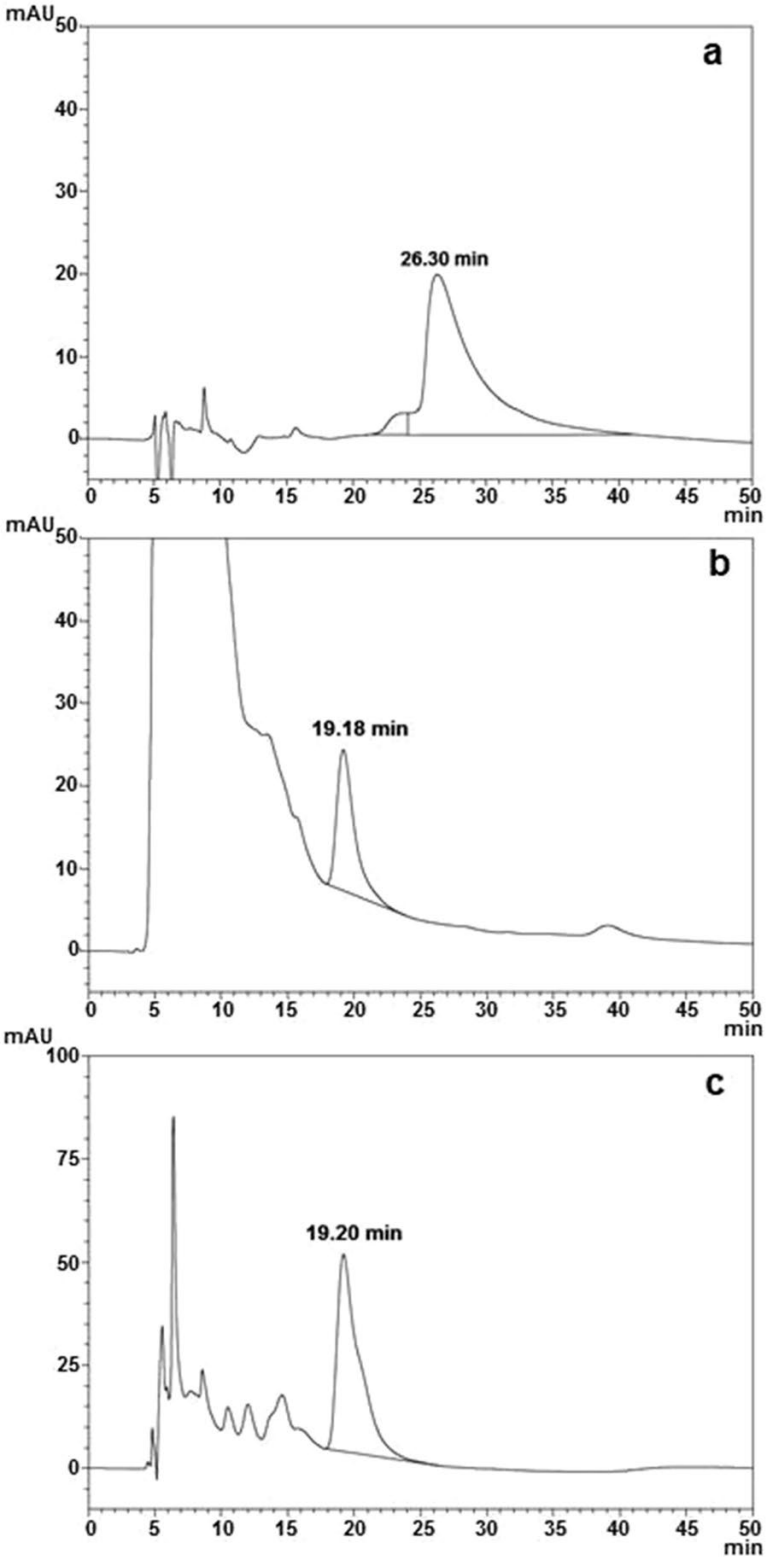
RP-HPLC analysis of: **a** hPRL internal standard with retention time (t_R_) of 26.30 min; **b** Δ_1-11_-G129R-hPRL, before purification from periplasmic fluid, with t_R_ of 19.18 min; **c** Δ_1-11_-G129R-hPRL after the two-step purification process, with t_R_ = 19.20. A C4 Vydac 214TP54 column (25 cm × 4.6 mm ID) was used. The mobile phase consisted of 71% 50 mM Tris–HCl buffer, pH 7.5, and 29% n-propanol, with a flow rate of 0.5 ml/min, column temperature maintained at 45 °C, monitoring at 220 nm and applying a sample volume of 10 µL (**a**) or 100 µL (**b, c**)

The analysis of the periplasmic fluid carried out before purification showed a retention time (t_R_) of 19.18 min for the Δ_1-11_-G129R-hPRL antagonist, which is lower than that of hPRL (26.30 min), indicating a lower hydrophobicity (Fig. 3a, b). The hydrophobicity of Δ_1-11_-G129-hPRL was then confirmed via the same RP-HPLC, analyzing now the final purified product, without the presence of the large amount of material that appeared in the initial part of the chromatogram (Fig. 3c) and that might potentially have influenced the antagonist t_R_. As can be observed, the antagonist t_R_ of 19.20 min was perfectly maintained even after purification (Fig. 3c).

### Purification of Δ_1-11_-G129R-hPRL from periplasmic fluid

**Fig. 4:**
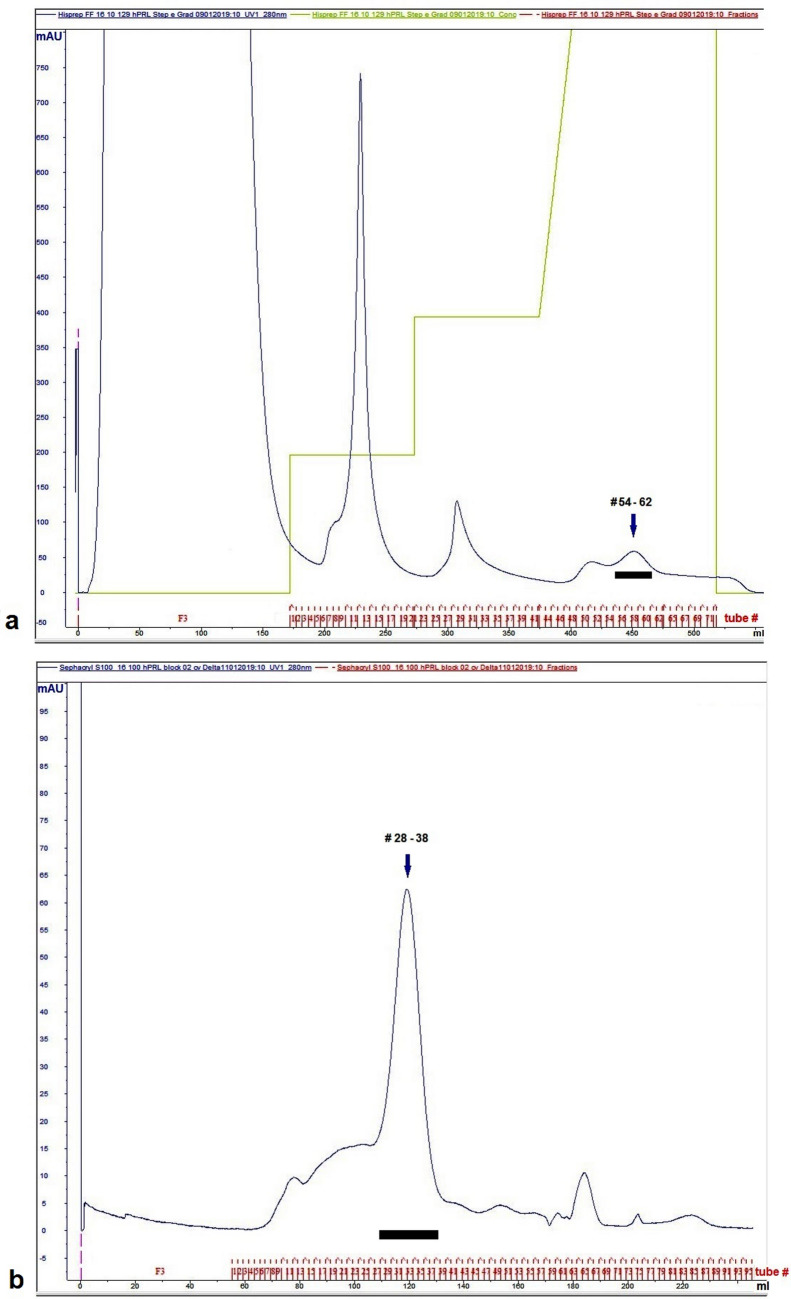
IMAC (**a**) and SEC (**b**) purification steps of Δ_1-11_-G129R-hPRL. The black bars correspond to the antagonist elution

The two-step purification was developed using the periplasmic fluid from a 3 L culture of BL21(DE3)-derived Δ_1-11_-G129R-hPRL. The first step, using metal affinity chromatography (Hisprep Fast Flow 16/10), was carried out as described for G129R-hPRL (Furigo et al. 2019). The pool of fractions #54 to #62 (Fig. 4a) corresponded to 80 mM imidazole elution, as was the case of G129R-hPRL (Furigo et al. 2019). The pool containing Δ_1-11_-G129R-hPRL was used for the second purification step via size-exclusion chromatography (SEC, Sephacryl S-100), the final product being eluted in fractions #28 to #38 (Fig. 4b).

**Fig. 5:**
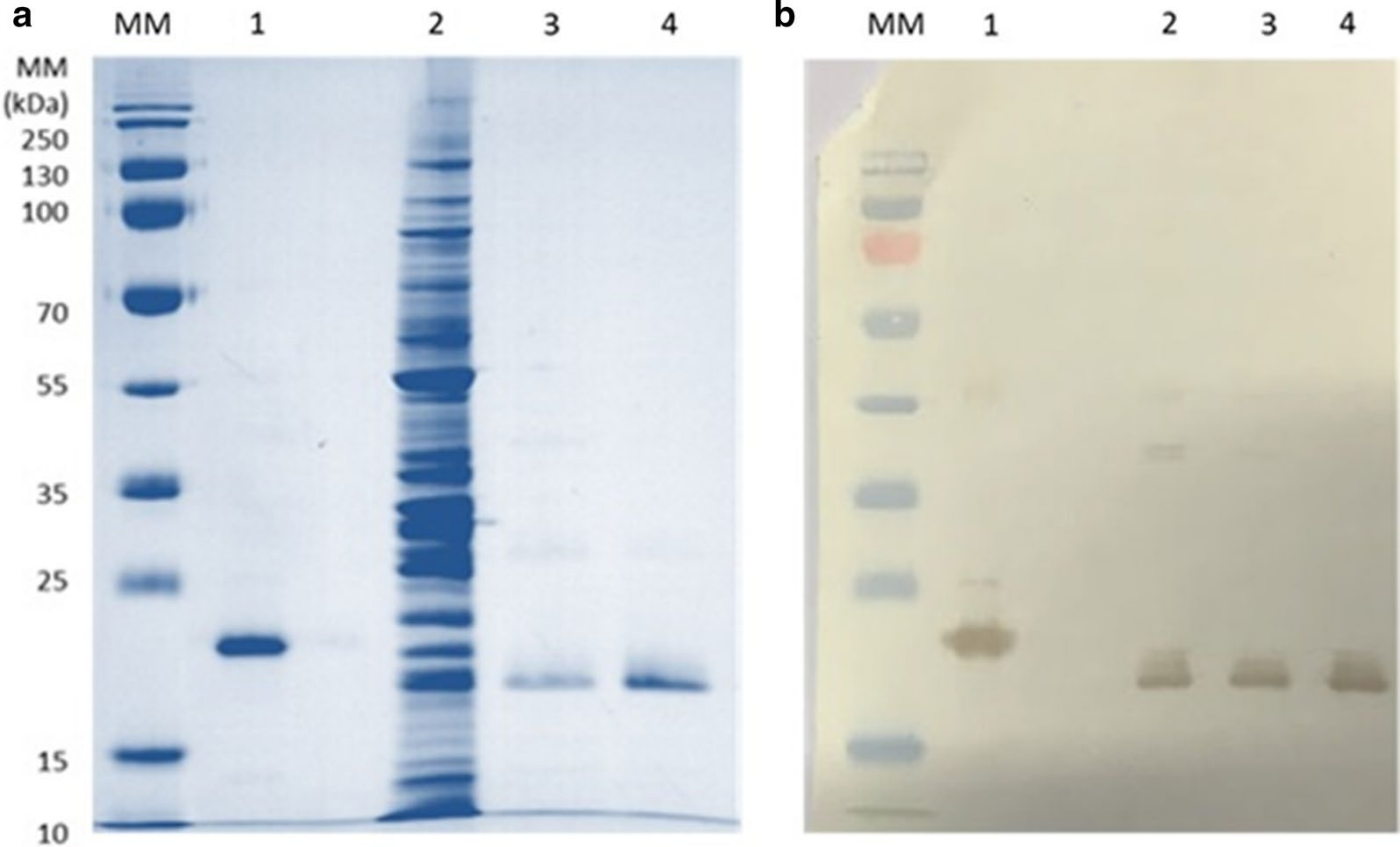
Comparative analysis of the chromatographic steps of Δ_1-11_-G129R-hPRL by SDS-PAGE under non-reducing conditions (**a**) and Western Blotting (**b**). (MM) molecular mass standards, (1) hPRL, (2) periplasmic fluid, (3) pool from IMAC and (4) pool from SEC

The SDS-PAGE (Fig. 5a) and Western Blotting (Fig. 5b) of samples before purification (lane 2) and after the two purification steps (lanes 3 and 4) showed the efficacy of the chromatographic techniques applied.

**Fig. 6:**
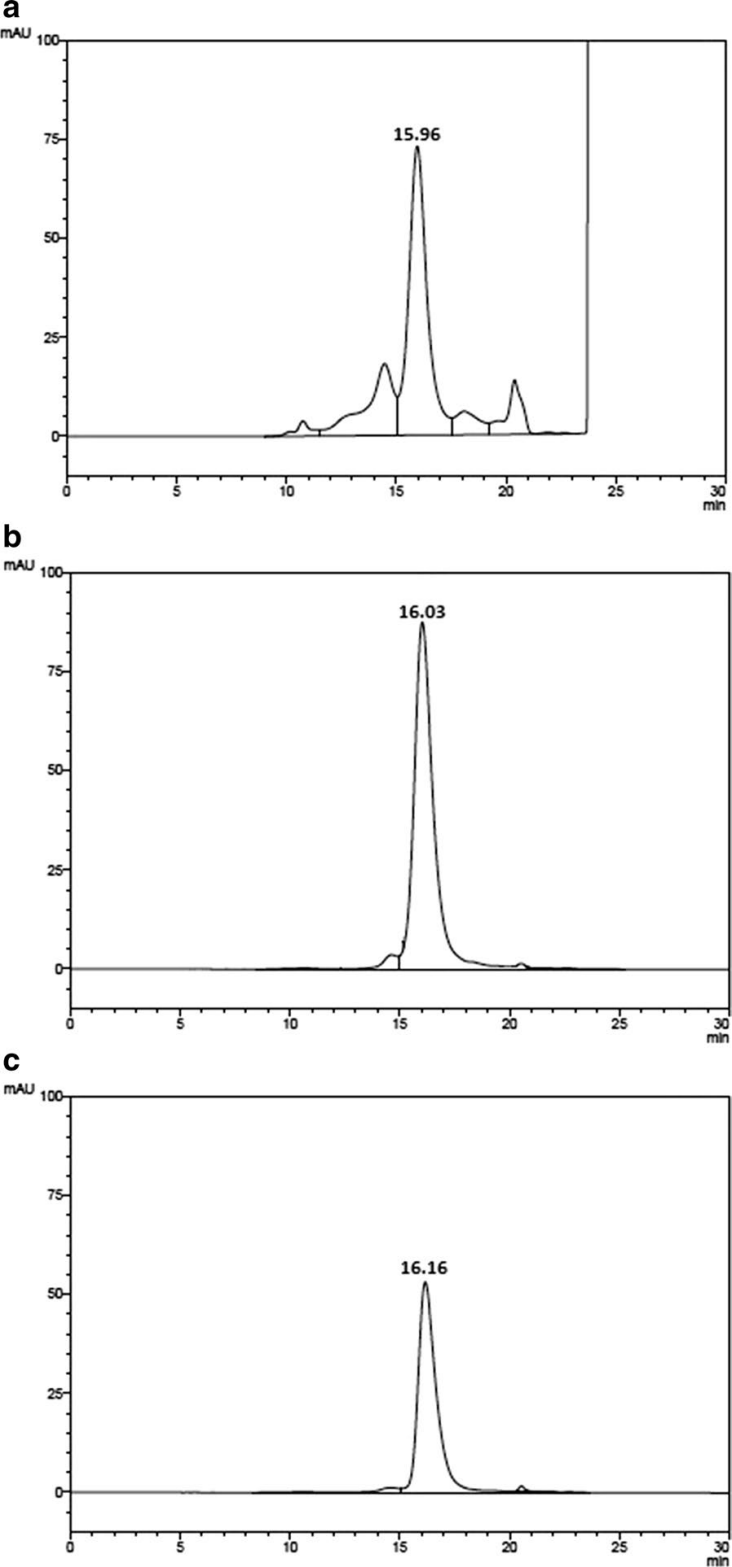
HPSEC analysis of the pool of Δ_1-11_-G129R-hPRL collected from IMAC (**a**) and from SEC (**b**) and of the internal standard of hPRL (**c**)

Most of periplasmic proteins were eliminated just after the first purification step, as confirmed by HPSEC analysis (Fig. 6a): the main peak, with t_R_ 15.96 min, corresponds to Δ_1-11_-G129R-hPRL and four contaminants were also still present. The final product, obtained by elution of the main peak, showed > 95% purity by HPSEC analysis, with a t_R_ = 16.03 min (Fig. 6b). The area under the peak of hPRL was used for quantification (Fig. 6c). A volumetric yield of 0.54 µg/mL antagonist, with purity above 95%, was obtained.

Table 1 shows the recovery and purity after each purification step, starting from a 3 L culture broth.

**Table 1:** Recovery and purity of Δ_1-11_-G129R-hPRL after each purification step

Purification step	Δ_1-11-_G129R-hPRL (μg)	Recovery (%)	Purity (%)
Periplasmic fluid	3600	–	–
IMAC (Hisprep FF)	1160	32.2	65.7
SEC (Sephacryl S100)	884	76.2	96.5

**Fig.7:**
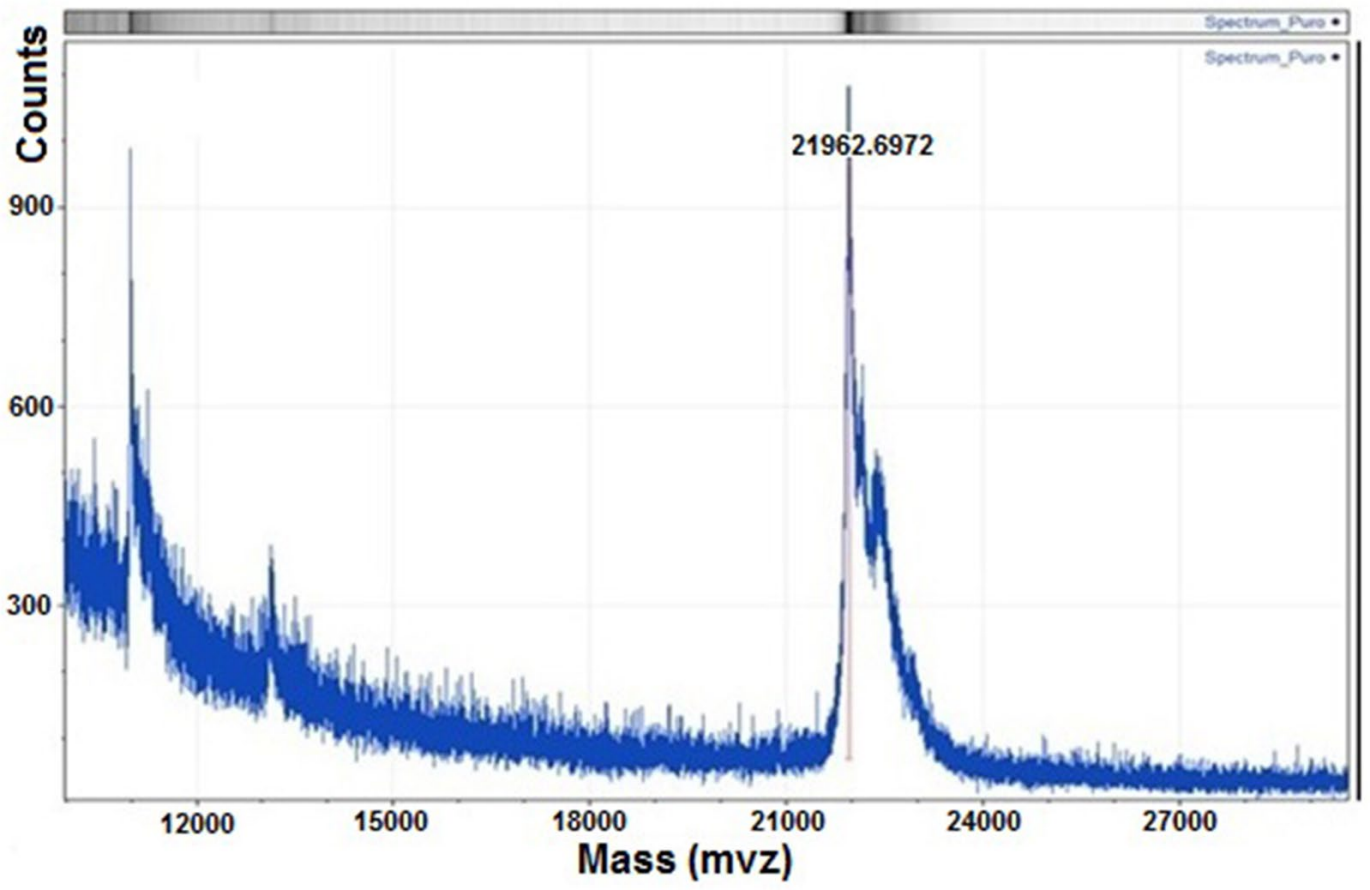
Example of the MALDI-TOF–MS analysis of purified Δ_1-11_-G129R-hPRL

MALDI-TOF–MS was used to confirm the theoretical molecular mass (MM) of Δ_1-11_-G129R-hPRL: 21,958.03 Da. Two independent assays determined a MM of 21,963.48 Da (+ 0.025%) and 21,952.59 Da (− 0.029%) (Fig. 7), confirming the high accuracy offered by this methodology.

### N-terminal data

The eight N-terminal amino acids of the Δ_1-11_ G129R-hPRL were determined as QVTLRDLF, lacking the two cysteines of the first disulfide bond (C4–C11) of human prolactin LPICPGGAARCQVTLRDLF.

### In vitro biological activity determination

**Fig. 8:**
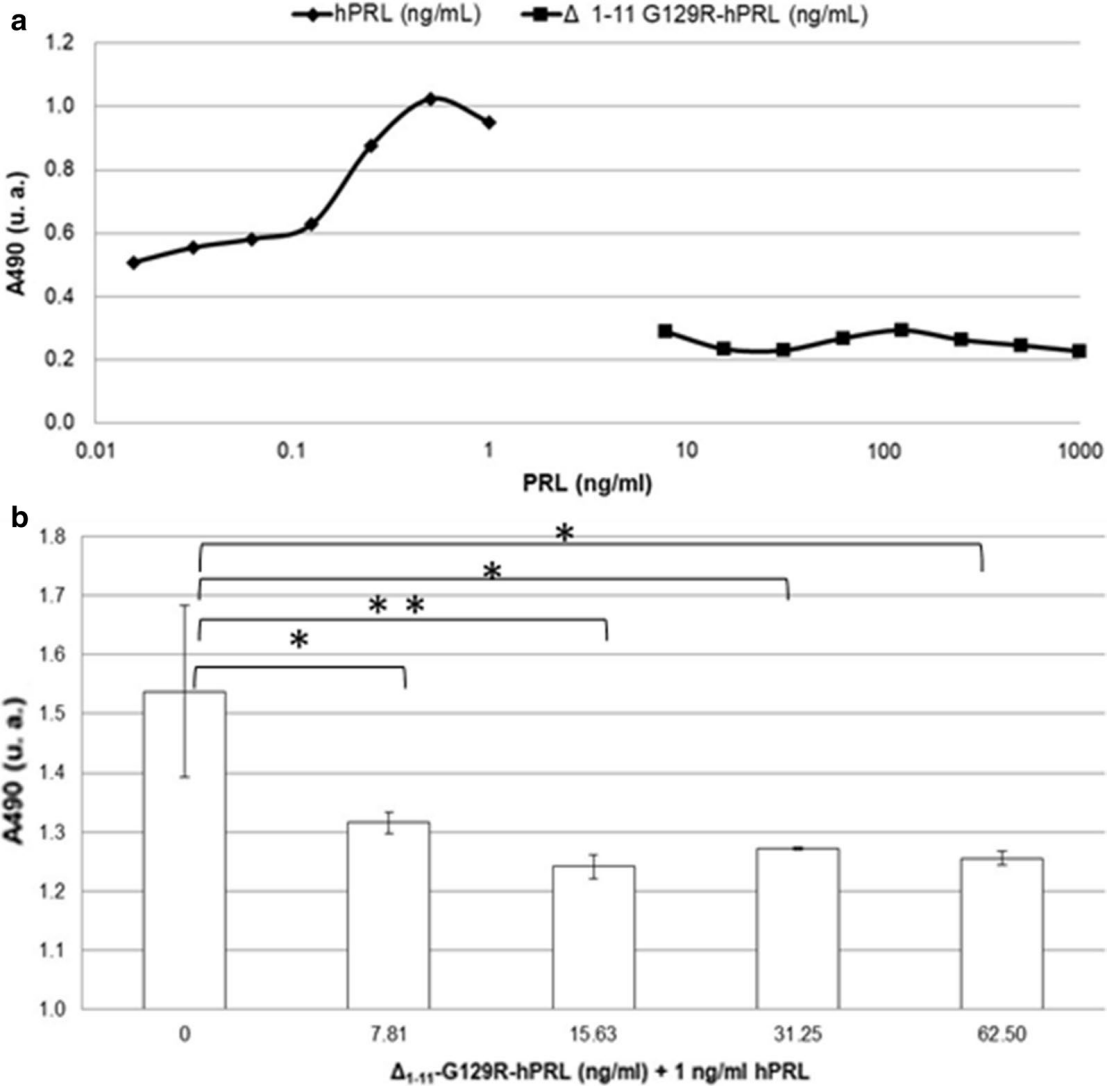
Agonistic (**a**) and antagonistic (**b**) effects of Δ_1-11_-G129R-hPRL in the proliferation assay based on Ba/F3-LLP cells. When the antagonist was added, the difference relative to the control was always significant: *P < 0.02; **P < 0.05

The Ba/F3-LLP proliferation assay based on mouse lymphoblastic cells confirmed the null agonistic effect of Δ_1-11_-G129R-hPRL up to 1000 ng/mL (Fig. 8a), while the antagonistic effect of this molecule was always significant, starting from a concentration of 7.8 ng/mL in the presence of 1 ng/mL of hPRL (Table 2) and reaching a value of ~ 23% with a ~ 16-fold molar excess of the antagonist (Fig. 8b).

**Table 2:** Antagonistic effect of different concentrations of Δ_1-11_-G129R-hPRL, in the presence of 1 ng/mL of hPRL, on Ba/F3-LLP cells proliferation

Δ_1-11_-G129R-hPRL (ng/mL)	Exp. 1 (A_490_)	Exp. 2 (A_490_)	Exp. 3 (A_490_)	Mean ± SD (A_490_)	CV (%)
0.00	1.71	1.46	1.45	1.54 ± 0.14	9.43
7.81	1.30	1.31	1.34	1.32 ± 0.02	1.35
15.63	1.26	1.24	1.22	1.24 ± 0.02	1.66
31.25	1.27	1.27	1.27	1.27 ± 0.01	0.18
62.50	1.26	1.26	1.24	1.26 ± 0.01	0.93

SD: standard deviation; CV: coefficient of variation

## Discussion

For the first time, the human prolactin receptor antagonist Δ_1-11_-G129R-hPRL has been synthesized, following also the tendency of our research group of giving priority to periplasmic expression. As far as we know this is the only hPRL antagonist ever synthesized in *E. coli* periplasm. The most studied prolactin antagonist is Δ_1-9_-G129R-hPRL (Ferraris et al 2013; Goffin 2017; Jomain et al. 2007; Oclon et al. 2018; Tallet et al. 2008), even though no clinical trial has been effectively carried out yet. The plasmid used in the present study was the same that should produce the prolactin antagonist G129R-hPRL without the initial nine residues. After MALDI-TOF–MS analysis and N-terminal amino acid determination, the absence of the eleven N-terminal residues was confirmed. We speculate that the difference between Δ_1-9_- and Δ_1-11_-G129R-hPRL could be due to a proteolytic processing at the amino termini that only occurs in the periplasm. A specific investigation of the synthesis of this new antagonist would therefore be particularly interesting. Several prolactin variants are found in human plasma with 22 kDa, 21 kDa, 16 kDa, 8 kDa and even 2 kDa and 1 kDa, produced via proteolytic cleavage by kallikrein, a trypsin-like serine protease, or by a cathepsin D-like protease (Ben-Jonathan et al. 1996; Sinha 1992). Although Δ_1-11_-G129R-hPRL antagonist was obtained here from *E. coli* periplasmic fluid, the cytoplasmic synthesis of human prolactin, presenting truncated N-termini up to the first 14 residues, did not show any undesired cleavage (Bernichtein et al. 2003a, b, c).

### Effects of temperature and IPTG concentration on the expression level

The specific expression of 0.16 μg/mL/A_600_ obtained for Δ_1-11_-G129R-hPRL at 35 °C was lower than that obtained for G129R-hPRL at 35 °C: 0.49 μg/mL/A_600_ (Furigo et al. 2019), and much lower than the hPRL expression of 0.93 μg/mL/A_600_ at 37 °C (Soares et al. 2008). Previous work on the periplasmic expression of the growth hormone antagonist G120R-hGH showed, in fact, that the highest specific productivity was obtained at 37 °C (1.34 μg/mL/A_600_) (Menezes et al 2017), differently from our maximum hGH production obtained at 42 °C by using the repressor gene pRK248 cIts: 3.9 μg/mL/A_600_ (Soares et al. 2003). For hPRL, the highest production was obtained at 37 °C (Soares et al. 2008), while for G129R-hPRL this was at 35 °C, using the lambda P_L_ promoter and the W3110 strain (Furigo et al. 2019). Thus, in our hands, periplasmic prolactin and its variants required, in general, a lower expression temperature than hGH. It is of note, moreover, that the periplasmic expression of hPRL and of its variants was generally quite problematic to the extent that the periplasmic production of the antagonist S179D-hPRL was insufficient for further studies (Ueda et al. 2009).

It is widely known that periplasmic expression has in general much lower yields (up to > 100-fold lower) than cytoplasmic expression. We can for example compare the hPRL cytoplasmic expression of 132.7 μg/mL/A_600)_ (Affonso et al. 2018) with the periplasmic expression of 0.93 μg/mL/A_600)_ reported by Soares et al. (2008) for the same hormone. Considering other authors, we should mention the production obtained in the cytoplasm by Goffin et al. (1994) for several hPRL analogues, that was of the order of 150 μg/mL. The quite low specific expression of our antagonist, mentioned above and obtained in erlenmayer flasks, can be greatly improved under controlled bioreactor conditions as reported by our research group in the case of hPRL production (Soares et al. 2008).

### Purification of Δ_1-11_-G129R-hPRL from periplasmic fluid

The first purification step of the present prolactin variant obtained from periplasmic fluid required 80 mM Imidazole. A lower molarity of 60 mM imidazole was used instead for hPRL elution in previous work (Ueda et al. 2001). The recovery in the IMAC step was lower than that previously obtained for hPRL: 84% (Ueda et al. 2001). The recovery from SEC was similar to that reported for G120R-hGH: 69% (Menezes et al. 2017).

### In vitro biological activity determination

Besides having shown no agonistic effect on mouse lymphoblastic cells proliferation, Δ_1-11_-G129R-hPRL antagonism versus hPRL (23%) was well above the one that we determined in previous work analyzing G120R-hGH (7%) (Menezes et al. 2017). Bernichtein et al. (2003a, b, c) reported for Δ_1-9_-G129R-hPRL or Δ_1-14_-G129R-hPRL a 50% antagonistic activity with a 100-fold antagonist molar excess or almost 100% with a 1000-fold molar excess, while Oclon et al. (2018) reported a 99% antagonist activity with a 100-fold molar excess of Δ_1-9_-G129R-hPRL. We suggest that the assay on the antagonistic effect should be standardized to a 1000-fold molar excess of antagonist as reported by Bernichtein et al. (2003a, b, c), to facilitate inter-laboratory comparisons.

Human PRL receptor is activated by prolactin, growth hormone and placental lactogens (Bernichtein et al 2003a, b, c). It is of note that, in the case of hPRL, even if the 13 first residues are removed, the hormone bioactivity is not affected in either the Ba/F3-LP or Nb2 rat cell assays (Jomain et al. 2007).

Since the full spectrum of functions of prolactin in health and disease, not only in humans, but in all vertebrates, is not completely understood (Bernard et al. 2019), the Δ_1-11_-G129R-hPRL may be important for further in vivo and in vitro studies. In view of clinical applications in the field of theranostic drug compounds, the binding of this molecule to iodine 131, gallium 67 or lutetium 177 could be useful for diagnosis and therapy. Δ_1-11_-G129R-hPRL should also be tested for the inhibition of cancer cell proliferation overexpressing the prolactin receptor (Cheal et al. 2018) or for the control of blood glucose levels in individuals with insulin resistance (Furigo et al. 2019). Its possible therapeutic applications will take advantage of having obtained this antagonist without an initial methionine and directly in its soluble and correctly folded form.

## Data Availability

Not applicable.
